# Intracytoplasmic Expression of IL-6 and IL-17A in Circulating CD4+ T Cells Are Strongly Associated with and Predict Disease Activity in Rheumatoid Arthritis: A Case-Control Study in Ghana

**DOI:** 10.1155/2020/2808413

**Published:** 2020-10-08

**Authors:** Samuel Asamoah Sakyi, Tonnies Abeku Buckman, Daniel Antwi-Berko, Kwame Yeboah-Mensah, Dzifa Dey, Eddie-Williams Owiredu, Benjamin Amoani, Richard Mantey

**Affiliations:** ^1^Department of Molecular Medicine, School of Medicine and Dentistry, Kwame Nkrumah University of Science and Technology, Kumasi, Ghana; ^2^Department of Basic and Applied Biology, University of Energy and Natural Resources, Sunyani, Ghana; ^3^Department of Medicine, School of Medicine and Dentistry, Komfo Anokye Teaching Hospital, Kwame Nkrumah University of Science and Technology, Kumasi, Ghana; ^4^Department of Medicine and Therapeutics, Korle-Bu Teaching Hospital, University of Ghana Medical School, Accra, Ghana; ^5^Department of Biomedical Sciences, College of Health and Allied Sciences, School of Allied Health Sciences, University of Cape Coast, Cape Coast, Ghana

## Abstract

**Background:**

T cell cytokines play important roles in the development and progression of rheumatoid arthritis (RA). Loss of Th1/Th2 and Th17/Treg balance has been reported in several inflammatory autoimmune diseases. However, their role in RA within hitherto rare Ghanaian context has not been explored. Here, we evaluated the intracytoplasmic CD4+ T cell cytokine patterns in rheumatoid arthritis patients in Ghana and determined their relationship with disease activity.

**Methods:**

This case-control study included 48 newly diagnosed RA patients and 30 apparent healthy controls from two major hospitals in Ghana. Validated structured questionnaires were administered to obtain demographic data; blood samples were collected and processed for flow cytometric analysis.

**Results:**

IFN-*γ*, TNF-*α*, IL-4, IL-6, IL-10, IL-17A, IL-6/IL-4, and IL-17/IL-10 expressions were significantly higher in RA cases compared to the healthy controls. The expression of IL-6 (0.00 (0.00-0.98) vs. 0.82 (0.34-1.10) vs. 1.56 (1.39-1.68), *p* < 0.0001), IL-17A (0.00 (0.00-0.02) vs. 0.19 (0.09-0.30) vs. 0.99 (0.64-1.25), *p* < 0.0001), and IL-17A/IL-10 (0.00 (0.00-0.39) vs. 0.15 (0.09-0.26) vs. 0.88 (0.41-1.47), *p* < 0.0001) increased significantly from the healthy controls through RA patients with low DAS scores to RA patients with moderate DAS scores. IL-6 (*β* = 0.681, *r*^2^ = 0.527, *p* < 0.0001), IL-17A (*β* = 0.770, *r*^2^ = 0.593, *p* < 0.0001), and IL-17A/IL-10 (*β* = 0.677, *r*^2^ = 0.452, *p* < 0.0001) expressions were significantly directly associated with DAS28 scores. IL-6 (cutoff = 1.32, sensitivity = 100.0%, specificity = 100.0%, accuracy = 100.0%, and AUC = 1.000) and IL-17A (cutoff = 0.58, sensitivity = 100.0%, specificity = 100.0%, accuracy = 100.0%, and AUC = 1.000) presented with the best discriminatory power in predicting moderate DAS scores from low DAS scores.

**Conclusion:**

Th1- and Th17-related cytokines predominate in the pathophysiology of RA, with IL-6 and IL-17 being principally and differentially expressed based on the severity of the disease. IL-6 and IL-17A could serve as useful prognostic and disease-monitoring markers in RA in the African context.

## 1. Introduction

Rheumatoid arthritis (RA) is the most common chronic, systemic, inflammatory autoimmune disorder with an estimated global prevalence of 1% [1]. The peak incidence usually occurs in individuals between the ages of 30 and 50 years and is characterized by persistent synovitis, pain, swelling, and progressive deterioration of the small joints of the hands and feet, accompanied by functional disability [2, 3].

The mechanisms underlying RA development and progression is complex, with T cells principally implicated in the pathophysiology of the disease [3, 4]. In RA, T helper (Th) cells facilitate B cell antibody production, induce macrophages development, and recruit other leukocytes to the sites of inflammation through their production of cytokines and chemokines [5]. Thus, Th cells play crucial roles in RA pathogenesis.

Th cell can differentiate into at least four distinct subpopulations comprising Th1, Th2, Th17, and T regulatory (Treg) cells. The Th cell subpopulations and the cytokines and chemokines they express and or produce act through diverse mechanisms that underpin the development and progression of RA. Evidently, imbalance Th1/Th2 and Th17/Treg cells have been implicated in RA development and progression [6–8]. However, the interplay of their cytokine pattern is minimally explored. Through their cytokine production, Th1 and Th17 cells play proinflammatory roles whereas Th2 and Treg cells play anti-inflammatory roles. Th1 cells produce interleukin- (IL-) 2, interferon-gamma (IFN-*γ*), and tumor necrosis factor-alpha (TNF-*α*) whereas Th2 cells produce mainly IL-4 and IL-5. Th2 cells also secrete IL-6, IL-10, and IL-13. Th17 cells secrete predominantly interleukin IL-17A and have recently been shown to be increased in the peripheral blood and synovial fluid of patients with RA [9–11]. Tregs produce IL-10 and transforming growth factor-beta (TGF-*β*) [12]. These cytokines play differential roles in RA [6, 8] and underscore the significance of cytokine patterns in RA development and the progression of the disease.

The outcomes of patients with RA have been related with the disease activity. Disease activity measures have thus been very valuable to reflect patient outcomes and response to therapy in clinical care and in clinical trials [13]. Although several measures exist to provide information about the various dimensions of outcomes, the American College of Rheumatology recommends the Disease Activity Score with 28-joint counts (DAS28) as one of the paramount, as it accurately reflects the disease activity; is sensitive to change; discriminates well between low, moderate, and high disease activity states; has remission criteria; and is feasible to perform in clinical settings [14].

RA was believed to be more common in developed countries; however, the disease is increasingly becoming more prevalent in developing countries, primarily due to improved diagnosis, adoption of westernized lifestyle, and increased access to health care [15–17]. Additionally, the burden of the disease in low- and middle-income countries has previously been under reported. In meta-analyses, Usenbo et al. [15] and Rudan et al. [16] indicated paucity of data on RA among the African population, highlighting the need for more RA-related research in the African context.

There is thus a dire need for more RA-related studies, especially within the African population. Against this background, we evaluated the intracytoplasmic CD4+ T cell cytokine patterns in rheumatoid arthritis patients in Ghana and determined their relationship with disease activity.

## 2. Materials and Methods

### 2.1. Study Design and Setting

This was a case-control study conducted between November 2015 and August 2017. Patient recruitment was done at the orthopedic units of Komfo Anokye Teaching Hospital (KATH) and Kumasi and Korle-Bu Teaching Hospital (KBTH), Accra, Ghana. KATH, the second largest hospital in Ghana, is a 1200-bed facility in the Kumasi Metropolis. Kumasi is the second major city in Ghana and has a projected population of 4,780,380. KBTH is the largest and third largest health facility in Ghana and Africa, respectively, with over 2000-bed capacity. The orthopedic units of both hospitals provide health care services to both in- and out-patients with rheumatologic and autoimmune conditions.

### 2.2. Participants Recruitment

A total of 48 consecutive consenting newly diagnosed RA patients, 29 from KATH and 19 from KBTH, were included as cases in this study. Diagnosis of RA at both clinics was based on the American College of Rheumatology/European League Against Rheumatism (ACR/EULAR) 2010 rheumatoid arthritis classification criteria by two independent Rheumatologists [18]. Apparent healthy blood donors with no chronic pain, cardiovascular complaints, chronic inflammatory diseases, malaria, TB, or parasitic infection and who gave informed consent were recruited and used as control. All patients were prednisolone-naive. Thirty healthy participants with no chronic pain, cardiovascular complaints, or other chronic inflammatory diseases were included as controls.

### 2.3. Ethics Approval and Consent to Participate

Ethical approval for this study was obtained from the Committee on Human Research, Publication and Ethics (CHRPE) of the School of Medical Sciences, Kwame Nkrumah University of Science and Technology (CHRPE/AP/003/16) and the institutional review board of KATH and KBTH. Written informed consent was obtained from all participants who opted to participate after the aims and objectives of the study had been explained to them. Participation was voluntary, and respondents were assured that the information obtained was strictly for research and academic purposes only and were guaranteed the liberty to opt out from the study at their own convenience.

### 2.4. Questionnaire Administration, Blood Pressure, and Anthropometric Evaluation

Questionnaires were administered to obtain sociodemographic data from the participants. Data collected include age, sex, marital, educational, and employment status. Additional clinical data relevant to the study were retrieved from the hospital's archives. Weight was measured in the upright position to the nearest 0.1 kg using a calibrated balance beam scale. Height was measured (subjects stood erect, barefoot, with feet together, looking forward) to the nearest 0.1 m using a measuring tape. Body mass index (BMI) was calculated using the equation [BMI (kg/m2) = weight/height^2^]. Blood pressure was measured with an automated blood pressure apparatus (Omron MX3-Omron Matsusaka Co., Ltd. Japan) from the right arm after the subject had been made to sit for at least five minutes. The average of the two readings taken five minutes apart was recorded.

### 2.5. Blood Sample Processing, Cell Preparation, and Flow Cytometric Analysis

Eight milliliters (8 ml) of venous blood was drawn from each RA patients and healthy controls. Four milliliters (4 ml) was dispensed into EDTA tubes for the estimation of erythrocyte sedimentation rate (ESR) using the Westergren method. The remaining 4 ml was dispensed into tubes containing heparin for the isolation of peripheral mononuclear cells (PBMCs) using the Ficoll-Paque density gradient centrifugation (Biochrom, Berlin, Germany). Briefly, the whole blood was poured into the Ficoll-containing tubes and centrifuged at 1500 rpm for 30 min at 4°C with no brakes. Cells were suspended in RPMI 1640 medium, supplemented with 0.5% DMSO and 0.5 ml Fetal Bovine Serum (Biochrom, Berlin, Germany) at a density of 0.5 × 10^4^ cells/ml. Collected PBMCs were stored at -80°C until further analysis. Prior to flow cytometric assessment of intracellular cytokine expression, frozen PBMCs were thawed in 37°C water bath for 15 min. The cells were washed in phosphate-buffered saline (PBS) and suspended in a small amount of PBS. Hundred microliters (100 *μ*l) of the resultant cell suspension was pipetted into 96-well plates, followed by centrifugation of plates at 1500 rpm for 5 min. About 180 *μ*l supernatant was pipetted and discarded after which cells were incubated for 10 min at room temperature in the dark. Cells were fixed and permeabilized using fixation/permeabilization reagent from BioLegend (San Diego, CA) followed by intracellular staining for CD4+ T cell expression of IFN-*γ*, TNF-*α*, IL-2, IL-4, IL-6, IL-10, and IL-17A using IFN-*γ* PE, TNF-*α* APC, IL-2 PerCP-Cy5.5, IL-4 APC, IL-6 PE, IL-10 APC, and IL-17A APC human antibodies. Cells were gated on lymphocyte population and CD4+ T cells, and analysis of percentage of cells expressing markers was done on the gated population using the Accuri C6 Flow Cytometer (Accuri Cytometers Inc., Ann Arbor, USA).

### 2.6. Assessment of Disease Activity

Disease activity was assessed based on the Disease Activity Score in 28 joints. Estimation was based on clinical parameters (tender and swollen joint counts), visual assessment scale, and laboratory markers of inflammation (erythrocyte sedimentation rate (ESR)). RA patients were grouped based on DAS28 scores; low disease activity (DAS28 ≤ 3.2), moderate disease activity (3.2 < DAS28 ≤ 5.1), and high disease activity (DAS28 > 5.1) [19].

### 2.7. Data Analysis

Flow cytometry data was analyzed with FlowJo 10.1.5 (FlowJo, LLC, USA). Statistical analysis was performed using the R Language for Statistical Computing version 3.6.0 [20]. Categorical data were presented as frequencies (percentages), and Chi square and Fisher's exact test statistic were used to test for association where applicable. For continuous data, normality was checked using the Shapiro-Wilk's test, as well as visual inspection with Q-Q plots. Normally distributed data were presented as mean ± SD, and significance of differences was assessed using independent *t*-tests. Nonparametric data were presented as median (interquartile ranges), and significance of differences was evaluated using the Mann–Whitney *U* tests and Kruskal-Wallis *W* with Dunn's multiple comparison tests, where applicable. The linear relationship between T cell cytokines (and their ratios) and DAS28 scores was assessed using linear regression models. Regression analysis was limited to cytokines with significantly different expression based on DAS28 subgroups. To determine the capacity of the cytokines to discriminate low and moderate DAS28 scores in RA, receiver operating characteristic (ROC) curve analysis was performed. The ROC curve analysis was based on binary logistic regression and discriminant classification analysis for low and moderate DAS28 groups. Analysis was restricted to controls, low and moderate DAS28 groups (we do not report data for high DAS28) because the relatively low number of RA patients with high DAS28 scores limited statistical reliability. All tests were two-sided, and *p* value < 0.05 was considered statistically significant.

## 3. Results

A total of 48 RA cases (mean age = 51.00 ± 13.01 years old) and 30 healthy controls (mean age = 47.47 ± 3.88 years old) were recruited for this study. There were more females than males, and a higher proportion of the participants were married and employed. The average ESR and DAS28 scores among the RA cases were 35.50 (29.25-55.25) mm/hr and 3.17 ± 1.07, respectively. The prevalence of low, moderate, and high DAS28 scores was 68.7%, 25.0%, and 6.3%, respectively (Table 1).

**Figure 1:**
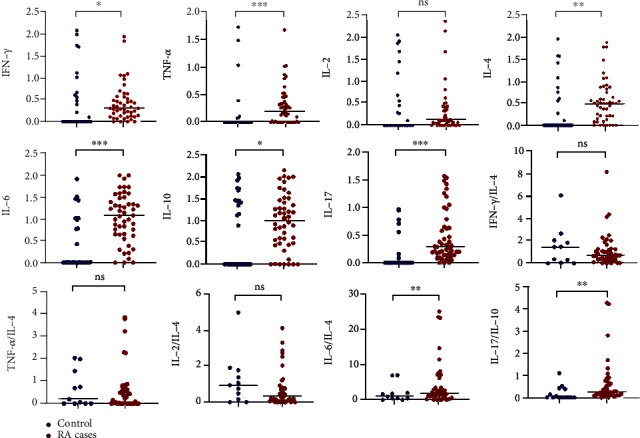
Comparison of T cell cytokines and their ratios between RA cases and healthy controls. ∗*p* < 0.05, ∗∗*p* < 0.01, ∗∗∗*p* < 0.0001.

IFN-*γ* (0.30 (0.13-0.55) vs. 0.00 (0.00-0.62), *p* = 0.032), TNF-*α* (0.20 (0.00-0.43) vs. 0.00 (0.00-0.02), *p* < 0.0001), IL-4 (0.49 (0.17-0.80) vs. 0.00 (0.00-0.64), *p* = 0.006), IL-6 (1.06 (0.63-1.43) vs. 0.00 (0.00-0.98), *p* < 0.0001), IL-10 (1.10 (0.58-1.49) vs. 0.01 (0.00-1.44), *p* = 0.045), IL-17A (0.29 (0.14-0.65) vs. 0.00 (0.00-0.02), *p* < 0.0001), IL-6/IL-4 (1.80 (0.67-4.84) vs. 1.00 (0.49-1.99), *p* = 0.018), and IL-17/IL-10 (0.23 (0.11-0.71) vs. 0.00 (0.00-0.39), *p* = 0.007) were significantly higher in RA cases compared to the healthy controls (Figure 1).

**Figure 2:**
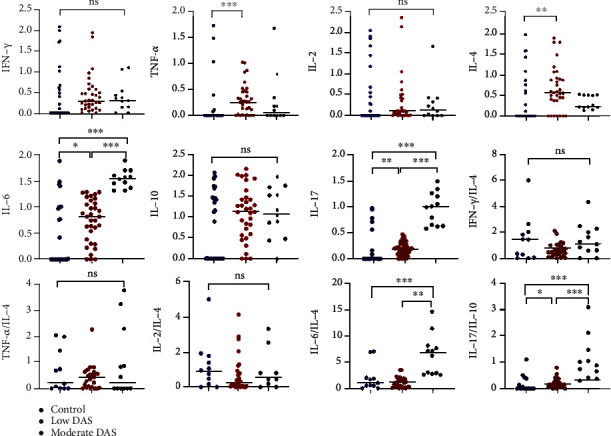
Comparison of T cell cytokines and their ratios between healthy controls and RA cases based on DAS28 scores. ∗*p* < 0.05, ∗∗*p* < 0.01, ∗∗∗*p* < 0.0001.

IL-6 (0.00 (0.00-0.98) vs. 0.82 (0.34-1.10) vs. 1.56 (1.39-1.68), *p* < 0.0001), IL-17A (0.00 (0.00-0.02) vs. 0.19 (0.09-0.30) vs. 0.99 (0.64-1.25), *p* < 0.0001) and IL-17A/IL-10 (0.00 (0.00-0.39) vs. 0.15 (0.09-0.26) vs. 0.88 (0.41-1.47), *p* < 0.0001) increased significantly from the healthy controls through RA patients with low DAS scores to RA patients with moderate DAS scores. RA patients with moderate DAS scores presented with significantly higher IL-6/IL-4 compared to RA patients with low DAS scores and the healthy controls, respectively. TNF-*α* and IL-4 were significantly higher in RA patients with low DAS scores compared to the healthy controls (Figure 2).

**Figure 3:**
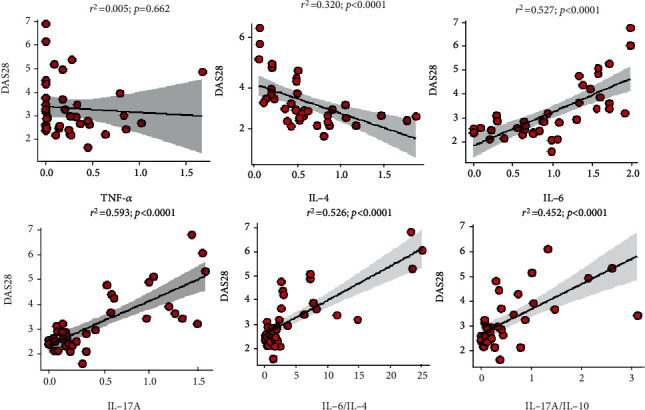
Linear relationship between T cell cytokines and their ratios and DAS28 scores among RA patients.

IL-6 (*β* = 0.681, *r*^2^ = 0.527, *p* < 0.0001), IL-17A (*β* = 0.770, *r*^2^ = 0.593, *p* < 0.0001), IL-6/IL-4 (*β* = 0.791, *r*^2^ = 0.526, *p* < 0.0001), and IL-17A/IL-10 (*β* = 0.677, *r*^2^ = 0.452, *p* < 0.0001) were significantly directly associated with DAS28 scores whereas IL-4 (*β* = −0.418, *r*^2^ = 0.320, *p* < 0.0001) was inversely related with DAS28 scores (Figure 3).

**Figure 4:**
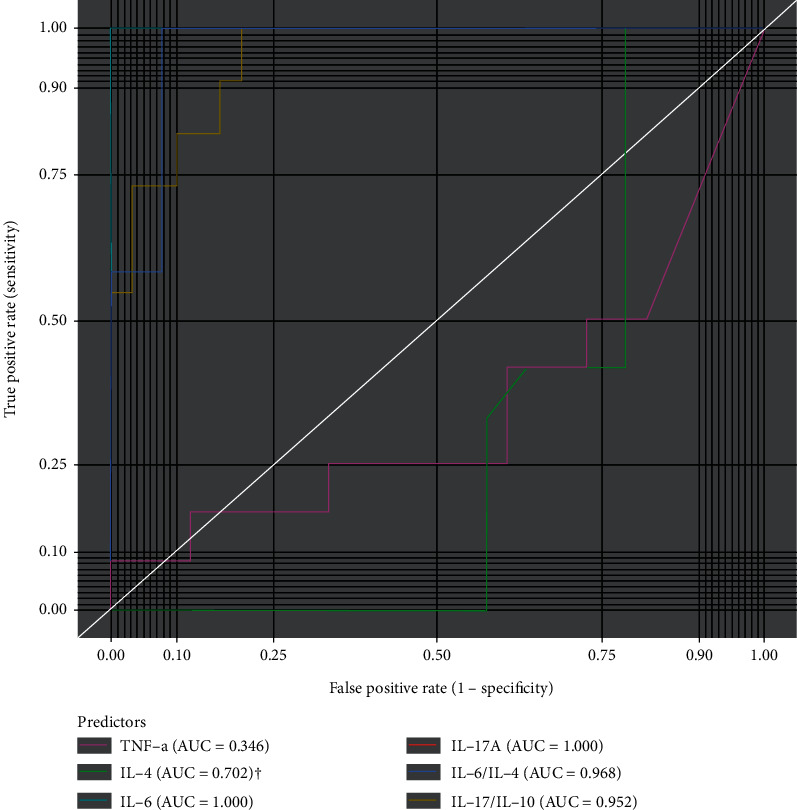
Performance of selected T cell cytokines to discriminate low and moderate DAS scores in RA. ^†^Test direction is negative (smaller test results indicate presence of condition).

IL-6 (cutoff = 1.32, sensitivity = 1.00 (0.71-1.00), specificity = 1.00 (0.87-1.00), accuracy = 100.0%, and AUC = 1.000), IL-17A (cutoff = 0.58, sensitivity = 1.00 (0.71-1.00), specificity = 1.00 (0.87-1.00), accuracy = 100, and AUC = 1.000), IL-6/IL-4 (cutoff = 2.54, sensitivity = 1.00 (0.71-1.00), specificity = 0.92 (0.75-0.99), accuracy = 94.74, and AUC = 0.968), and IL-17A/IL-10 (cutoff = 299, sensitivity = 1.00 (0.69-1.00), specificity = 0.80 (0.62-0.91), accuracy = 85.37, and AUC = 0.952) presented with the best discriminatory power in predicting moderate DAS scores from low DAS scores (Figure 4).

## 4. Discussion

Rheumatoid arthritis (RA) is a world-wide disease affecting adults with a global prevalence of 1%. Previous studies by Adebajo (1992), Harries (1962), and Bagg (1979) reported prevalence between 0.0 and 0.9% in Africa [21, 22]. In recent times, however, RA, a hitherto rare condition in Africa, has been observing increasing cases primarily due to improved diagnosis, adoption of westernized lifestyle, and increased access to health care. McGill observed an increasing frequency of RA and Systemic Lupus Erythematosus (SLE) among East, Central, and South Africa [23]. Adelowo et al. (2010), in a retrospective study in Nigeria, also reported that RA may not be rare and its presentation may not be much different from those in other populations [24]. Ampofo et al. (2016) in a cross-sectional study in Ghana reported 68% of study participants reported positive for RA [25].

In this comprehensive analysis of intracytoplasmic cytokine profile of circulating CD4+ T cells, we found that, with the exception of IL-2, all other cytokine expressions were significantly higher in RA compared to healthy controls. Of importance, IFN-*γ* and TNF-*α* expressions were significantly higher in RA, as expected. IFN-*γ* and TNF-*α* are proinflammatory cytokines produced by Th1 cells. IFN-*γ* stimulate inflammation in RA by facilitating macrophage activation and enhancing the activity of natural killer cells [1], and TNF-*α* exacerbates tissue damage by promoting other inflammatory cytokines such as IL-1 and IL-8 produced by macrophages, fibroblast, and synovial cells [26]. Our findings thus corroborate previous reports [1, 8] on the role of Th1 and the interplay of their cytokines in RA development.

Interestingly, IL-6 was also higher in RA compared to the healthy controls. Conventionally, Th2 cytokines are considered to have anti-inflammatory effector functions, and it was earlier thought, based on studies in murine models, that IL-6 was produced by Th2 cell; hence, should exhibit anti-inflammatory roles [27]. However, evidence suggests that, as opposed to murine models, in humans, IL-6 can be expressed by Th1 cells, hence not limited to the Th2 cells [8, 27]. Thus, the high IL-6 in RA suggests that its expression in CD4+ T cell in RA could be skewed towards the Th1 phenotype, hence, more associated with proinflammatory than anti-inflammatory effects. Indeed, evidence suggests that IL-6 is a pleiotropic cytokine with broad-ranging effects and acts in a context-dependent manner [28]. A study by Nakahara et al. also reported that IL-6 may not have a direct effect on synovial fibroblast and chondrocyte but improve the efficacy of TNF-*α* in RA [29].

We also observed an imbalance of Th17/Treg cytokines. IL-17A and IL-17/IL-10 expressions were higher in RA compared to the controls. Evidence suggests that IL-17A induces inflammation by mediating the secretion of other cytokines and chemokines [30]. In a study by van Hamburg et al. [31], Th17-producing cells induced secretion of IL-6, IL-8, and tissue-destructive enzymes, such as MMP-1 and MMP-3 by synovial fibroblasts. In another study, Niu et al. found an increase in peripheral Th17-related cytokines levels in RA compared with healthy controls, as consistent with our study findings. Interestingly, we also found the expression of IL-4 and IL-10 which are anti-inflammatory cytokines produced by Th2 and Treg cells, respectively, to be higher in RA than the controls. The higher expression of IL-10 in RA could be due to possible compensatory mechanism initiated to ameliorate the inflammation in the early phases of the disease, as evidenced by the higher IL-4 and IL-10 expression in RA patients with low DAS28 compared to controls, but not in patients with high DAS28 scores. Together, these findings substantiate the predominance of Th1- and Th17-related cytokines in the pathophysiology of RA.

In contrast with a study by Chen et al. [1], we found no significant association between DAS28 and IL-2; however, IL-6, IL-17A, and IL-17A/IL-10 increased from the healthy controls to RA patients with low DAS28 to patients with moderate DAS28 scores, suggesting that IL-6, IL-17A, and the Th17/Treg axis could be the key drivers of the progression of RA among the study population. We could not comment on the dynamics of these markers in patients with high DAS28 because only three RA patients had high DAS28 scores. It is possible that Africans could be prone to less severe disease; however, this is only a supposition given the limited data on RA in the context of Africa, and warrants further research. On the other hand, the potential roles of IL-6, IL-17A, and the Th17/Treg axis as drivers of RA progression is confirmed by the strong linear association between IL-6, IL-17A, and IL-17A/IL-10 with DAS28 scores. This finding is partly consistent with a study by Li et al. [32] who found a direct relationship between IL-17A and DAS28 scores in rheumatoid arthritis patients. Indeed, high IL-6 and IL-17 have been implicated in higher DAS28 scores, which together correlate with radiographical progression of RA patients [33–36]. Nonetheless, contrary to our study, Chung et al. [37], using enzyme-linked immunosorbent assay, reported no significant correlation between IL-6 and DAS28 scores. We attribute this discrepancy to the limited scale of RA subjects and differences in methods used in assessing cytokines.

The strong linear relationships between IL-6, IL-17A, IL-6/IL-4, IL-17A/IL-10, and DAS28 scores suggest that these cytokines could be useful in discerning the severity of the disease. To test this, we assessed the capabilities of the cytokine patterns to discriminate between disease activities by using ROC curve analysis with reference to low DAS28 score. Among these, IL-6 and IL-17A presented with the best discriminatory power with excellent sensitivity, specificity, and accuracy in predicting moderate DAS28 scores from low DAS28 scores, followed by IL-6/IL-4 and IL-17A/IL-10. In a study by Baillet et al., IL-6 was identified as a surrogate marker of synovial inflammation at baseline, and repeated measurements were a factor for structural progression in early RA among French early arthritis cohort [34]. In another study by Boyapati et al., high baseline IL-6 adequately identified a subgroup of RA patients with rapid joint damage and clinical progression [38]. In a prospective study by Kirkham et al., IL-17 mRNA expression was found to be predictive of joint damage progression in RA [39]. A study by Moran et al. also found that IL-17A is highly expressed in the inflammatory joint and drives disease activity in RA [40]. The limitation of the study is that eosinophilia mostly due to parasitic infections has effect on IL-17; however, the current study did not consciously look out for eosinophilia among cases but only inferred from questionnaires administered. Collectively, however, our findings, together with previous reports, confirm the pivotal roles played by IL-6 and IL-17A in the progression of the disease and highlight the potential prognostic and disease-monitoring applicability of IL-6 and IL-17A in RA.

## 5. Conclusion

Th1- and Th17-related cytokines predominate in the pathophysiology of RA, with IL-6 and IL-17 being principally and differentially expressed based on the severity of the disease. IL-6 and IL-17A could serve as useful prognostic and disease-monitoring markers in RA in the African context.

## Figures and Tables

**Table 1:** Demographic and clinical characteristics of the study population.

Variables	RA cases (*n* = 48)	Control (*n* = 30)	*p* value
Age (years)^†^	51.00 ± 13.01	47.47 ± 3.88	0.083
Sex			0.062
Male	8 (17.0)	11 (36.7)	
Female	39 (83.0)	19 (63.3)	
Marital status			0.641
Single	18 (37.5)	13 (43.3)	
Married	30 (62.5)	17 (56.7)	
Educational status			0.021
No education	2 (4.2)	3 (10.0)	
Primary	13 (27.1)	9 (30.0)	
Secondary	25 (52.1)	6 (20.0)	
Tertiary	8 (16.7)	12 (40.0)	
Employment status			0.043
Unemployed	10 (20.8)	1 (3.3)	
Employed	38 (79.2)	29 (96.7)	
BMI (kg/m^2^)^†^	30.23 ± 7.24	27.65 ± 5.12	0.093^†^
SBP (mmHg)^†^	129.35 ± 13.63	128.90 ± 6.88	0.846^†^
DBP (mmHg)^†^	82.60 ± 9.74	82.83 ± 5.17	0.893^†^
ESR (mm/hr)^†^	35.50 (29.25-55.25)	na	na
DAS score^†^	3.17 ± 1.07	na	na
Low	33 (68.7)	na	na
Moderate	12 (25.0)	na	na
High	3 (6.3)	na	na

^†^Mean ± SD and compared with independent *t*-test where applicable; BMI: body mass index; SBP: systolic blood pressure; DBP: diastolic blood pressure; ESR: erythrocyte sedimentation rate.

## Data Availability

The datasets used and/or analyzed during the current study are available from the corresponding author on reasonable request.
